# Perspective of an advocate: best practices for Community Outreach Advocacy

**DOI:** 10.1186/1750-9378-8-S1-S3

**Published:** 2013-07-15

**Authors:** Emmanuel J Kandusi

**Affiliations:** 1 Tanzania 50 Plus Campaign, Tanzania

## Abstract

Of all the different types of advocacy, Community Outreach Advocacy (COA) is the best methodology. This methodology is the prolegomena to the achievement of all other advocacies including political, fundraising, research, support and education. This is because the outcome of COA awareness work is an informed group of people. Such people can comprehend and tackle any other initiatives in addressing the issue at stake and so prevent and control *unnecessary suffering and deaths* caused by *cancer!* At the end of the day, the African Cancer Advocates Consortium (ACAC) will achieve its mission to ***“Make Cancer a Top Priority in Africa”*** through informed people.

## Background

“*Cancer is a group of diseases characterized by uncontrolled growth and spread of abnormal cells. If the spread is not controlled*, *it can result in death. Cancer is caused by both external factors* (*tobacco*, *infectious organisms*, *chemicals*, *and radiation*) *and internal factors* (*inherited mutations*, *hormones*, *immune conditions*, *and mutations that occur from metabolism*)*” *[1]. Cancer is increasingly a critical public health problem in Africa. Similar to the Bible quote of *“My people are destroyed for lack of knowledge” *[2], the high level of cancer morbidity and mortality in Africa is highly due to ignorance of this disease in African communities. In order to increase public knowledge and awareness about cancer, we need to come up with a deliberate plan of action to combat it. Community Outreach Advocacy (COA) offers the best advocacy practice that can be replicated in diverse communities. However, COA must be evidence-based and built on data that accurately and completely capture the occurrence, causes, prevention, and treatment of cancer in all African populations [3].

## Cancer in Africa

“*Cancer is a serious problem in the developing world*, *including Africa*, *and threatens to rival communicable diseases in terms of morbidity and mortality in the coming decades. While infectious diseases remain critical problems in Africa*, *it is also clear that increases in the rate of cancer in Africa are of serious concern. The World Cancer Report 2010 estimates that cancer in sub-Saharan Africa will increase by 50-75% by 2020. With increasing westernization of lifestyle*, *increased smoking*, *extension of life expectancy*, *and decreases in deaths from communicable diseases*, *cancer promises to be an increasingly important problem in Africa.*”[4]

“*Cancer kills more people each year than HIV/AIDS*, *malaria and tuberculosis combined.*” [5] “*Cancer killed 7.6 million people in 2005. By 2020 it could kill 16 million people a year. More than 75% of new cancer cases and cancer deaths will be in the developing world*, *mostly Africa Sub-Sahara. More than 70% of cancers in developing countries are diagnosed too late for cure. The remaining 25% will occur in developed countries.*”[5] The low rate of deaths in developed countries is due to the heavy investment in cancer control, cancer care and cancer research fostered by their governments, private sector and philanthropists. For example, there are many advanced patient advocacy organizations in North America and Europe. Both continents also have specific media outlets dedicated to cancer education and cancer testing easily available to all who need it. While the developed countries can afford to *walk* in the fight against cancer, developing countries in Africa must *run*!

## Community outreach advocacy in Africa

Africa should come up with deliberate implementable action plan which can make a difference to stemming the increase of cancer rates, and aid with prevention and control. While we have the projections on the rise of cancer incidences in Africa from respected international agencies, it is up to us to stop cancer in its track. African governments need to make cancer a *top priority* in their countries. Efforts they have had on malaria, tuberculosis and HIV/AIDS campaigns should be replicated for cancer campaigns. Africa should immediately invest heavily in cancer prevention, modern cancer treatment and cancer research. The Ministries of Health in all African countries should come-up with a *National Multi- Sectoral Strategic Framework on Cancer*, a policy document for all cancer advocacy work. In addition, the National Cancer Control Policies of each African country should be inclusive of all stakeholders – “*including individuals*, *families*, *and local and international communities*, *intergovernmental organizations and religious institutions*, *civil society*, *academia*, *media*, *voluntary association*, *and where and as appropriate*, *the private sector and industry*, *in support of national efforts for cancer prevention and control.*” [6] This will lead to a multi-sectoral, holistic, government-centric and society engaged initiative for the prevention and control of cancer in Africa. Prevention and control of cancer related diseases must be the corner-stone of all initiatives addressing cancer in Africa. While improving prevention, treatment and research, it is imperative that the public should be educated on cancer issues. The primary focus of community outreach in Africa should include:

**• *To eradicate tobacco consumption.*** Tobacco remains the most important risk factor for cancer. In the 20th century, approximately 100 million people die world-wide from tobacco-associated diseases.

**• *Encourage healthy lifestyle and natural diet.*** Outreach efforts should include educating people to avoid red meat and high-fat dairy products. Also, frequent consumption of fruit and vegetables and physical activity can make a big difference in preventing cancer.

**• *Early detection through testing*,** particularly for prostate, cervical and breast cancers, allow for successful treatment and cure. The earlier cancer is detected, the more easily and effectively it can be treated.[7]

African public need to be well informed on the risk factors for cancer and educated on the value of testing / screening for early detection of cancer. People should not wait for warning signs or symptoms of cancer. People should be made aware that prevention is better than cure and that a cancer diagnosis in the early stage is better than the late stage. *In most cancers*, *the earlier it is discovered the better chance of cure.* By acting now, Africa can achieve significant reductions in cancer cases, morbidity and mortality by the year 2020.

## Best practices for community outreach advocacy

The art of Community Outreach Advocacy is imperative in Africa to enable our interlocutors have timely opportunities to reduce incidences, suffering and deaths caused by cancers. In order for Africa to be very effective in its endeavor, it must adhere to best practices for Community Outreach Advocacy which include but are not limited to the following recommendations:

### Conduct a Situational analysis

It is imperative to conduct a needs assessment or baseline survey. This will enable an understanding and assessment of the target community health assets and needs. In turn, it will be possible to establish a database of current resources.

### Develop a Community Outreach Advocacy strategic plan of action

Community Outreach Advocacy should incorporate outreach, identifying cases through screening, palliation and pain management, holistic health and patient/healthcare consumer rights based approach. Advocates should develop and organize a guidebook with **SMART** objectives based on available community data that is [8]:

• **S**pecific - who/what?

• **M**easurable – how much?

• **A**chievable within a time frame and available resources.

• **R**ealistic

• **T**ime-phased, including completion time and/or when the objective will be measured.

### Establish collaboration and networking

Advocates should not work alone and should partner with the government and other advocates to make outreach a societal effort. In addition, it is important to establish and maintain: (i) partnerships with cancer treatment centers in targeted communities, including public hospitals, private hospitals, and hospices; (ii) mechanisms to tap mainstream cancer awareness groups and activities; (iii) mechanisms to engage public officials or politicians for global resonance to generate direction, dialogue, preparedness and action on cancer at the highest political level; (iv) collaborations with researchers and academic institutions; (v) linkage with chronic points of care such as health facilities, cancer wards, and social service departments for children and senior citizens; (vi) linkage with pharmaceutical companies and private companies to tap into their corporate platform to provide support for cancer prevention and control.

### Partner with the media

Advocates should also establish partnership with the media to support their community cancer awareness and prevention sensitization through television and radio programs, magazine and newspaper articles.

### Holistic approach to outreach

A holistic approach should be used for outreach, including tailored and bi-directional dialogue with targeted communities to ensure that the outreach program is community centered, empowering and community-centric.

## Conclusion

Our African Cancer Advocates Consortium (ACAC) mission is to ***“Make Cancer a Top Priority in Africa”****!* To fulfill this mission, Africa is challenged to intensify community outreach advocacy in collaborative partnership with other advocacy programs including political, fundraising, research, support and education. The opportunity to achieve our mission has been availed to us. It is however up to us to put to praxis all advocacy work we had consensus on during our 2011 inaugural meeting in Cairo, Egypt. Let us get started – *Together we can succeed* – Saving other peoples’ lives is saving our own lives!

## Abbreviations

**ACAC:** African Cancer Advocates Consortium; **AIDS:** Acquired Immune Deficiency Syndrome; **CHRP:** Centre for Human Rights Promotion, **COA:** Community Outreach Advocacy; **HIV:** human immunodeficiency virus**; NCD:** Non Communicable Disease; **PSA:** Prostate Specific Antigen; **SMART: S**pecific **M**easurable **A**chievable **R**ealistic **T**ime-phased.

## Competing interests

The author declares to have no competing interests.

## Declarations

The authors declare that funding for publication of the article was funded by an award provided by the National Cancer Institute (Award Number R13CA162899).

This article has been published as part of *Infectious Agents and Cancer* Volume 8 Supplement 1, 2013: Selected articles from The International Workshop on Cancer Advocacy for African Countries (CAAC) 2011. The full contents of the supplement are available online at http://www.infectagentscancer.com/supplements/8/S1 The Supplement Editors declare they have no competing interests.
